# Reduced tumorigenicity and pathogenicity of cervical carcinoma SiHa cells selected for resistance to cidofovir

**DOI:** 10.1186/1476-4598-12-158

**Published:** 2013-12-10

**Authors:** Tim De Schutter, Graciela Andrei, Dimitri Topalis, Sophie Duraffour, Tania Mitera, Joost van den Oord, Patrick Matthys, Robert Snoeck

**Affiliations:** 1 Rega Institute, Laboratory of Virology and Chemotherapy, KU Leuven, Leuven, Belgium; 2 Rega Institute, Laboratory of Immunobiology, KU Leuven, Leuven, Belgium; 3 Laboratory of Morphology and Molecular Pathology, UZ Leuven, Leuven, Belgium; 4 Rega Institute for Medical Research, KU Leuven, Minderbroedersstraat 10, B-3000 Leuven, Belgium

**Keywords:** Cidofovir, Cervical cancer, Human papillomavirus, Xenografts, Inflammatory response, Microarrays

## Abstract

**Background:**

Insights into the mechanisms associated with chemotherapy-resistance are important for implementation of therapeutic strategies and for unraveling the mode of action of chemotherapeutics. Although cidofovir (CDV) has proven efficacious in the treatment of human papillomavirus (HPV)-induced proliferation, no studies concerning the development of resistance to CDV in HPV-positive tumor cells have been performed yet.

**Methods:**

From the cervical carcinoma SiHa cells (SiHa_*parental*_), which are HPV-16 positive, cidofovir-resistant cells (SiHa_*CDV*_) were selected, and differential gene expression profiles were analyzed by means of microarrays. We examined *in vitro* phenotyping of resistant cells compared to parental cells as well as tumorigenicity and pathogenicity in a mouse-xenograft model.

**Results:**

SiHa_*CDV*_ had a resistant phenotype and a reduced growth both *in vitro* and *in vivo*. A markedly diminished inflammatory response (as measured by production of host- and tumor-derived cytokines and number of neutrophils and macrophages in spleen) was induced by SiHa_*CDV*_ than by SiHa_*parental*_ in the xenograft model. Gene expression profiling identified several genes with differential expression upon acquisition of CDV-resistance and pointed to a diminished induction of inflammatory response in SiHa_*CDV*_ compared to SiHa_*parental*_.

**Conclusions:**

Our results indicate that acquisition of resistance to cidofovir in SiHa cells is linked to reduced pathogenicity. The present study contributes to our understanding on the antiproliferative effects of CDV and on the mechanisms involved, the inflammatory response playing a central role.

## Background

Three acyclic nucleoside phosphonate analogues (ANPs), i.e. tenofovir (PMPA), adefovir (PMEA) and cidofovir (CDV), are approved for the treatment of viral infections [1,2]. Tenofovir and adefovir are active against retroviruses and hepadnaviruses, their oral prodrug forms being approved for therapy of HIV (PMPA) and of chronic hepatitis B virus infections (PMPA and PMEA). Although CDV is formally licensed for treatment of cytomegalovirus retinitis in AIDS patients, it is often used off-label for the management of diseases caused by several DNA viruses, including adeno-, pox-, papilloma-, polyoma-, and herpesviruses others than cytomegalovirus [3-6].

Besides their well-recognized antiviral properties, some ANPs have shown anticancer potency. For instance, PMEA, PMEDAP, PMEG, and prodrugs of PMEG [i.e. cPr-PMDEDAP, GS-9219 and GS-9191] showed marked cytotoxic properties *in vitro*[7-9]. Additionally, *in vivo* antitumor activities for these compounds have been described in different animal models: GS-9219 in a pet dog model of non-Hodgkin's lymphoma [10] and cPr-PMEDAP in a rat choriocarcinoma tumor model [11]. A close correlation between the cytostatic activities of PME derivatives and the inhibitory effects of their active metabolites (diphosphate forms) on cellular DNA polymerases α, δ, and ϵ has been established. In these studies, PMEG-diphosphate (PMEGpp) emerged as the most potent chain-terminating inhibitor of cellular DNA polymerases [12,13]. The utility of PMEG as an anticancer agent is limited by poor cellular permeability and toxicity [13,14] and prodrugs, such as GS-9191 and GS-9219, were designed to increase the permeability and accumulation of PMEGpp in the cells [10,13].

Cidofovir represents also an ANP with marked antiproliferative effects but unlike PMEG, the effects of CDV-diphosphate (CDVpp) on cellular DNA polymerization are weak [inhibition constant (Ki) of CDVpp for cellular DNA polymerase-α of 51 μM *versus* 0.55 μM for PMEGpp]. In addition, CDVpp is not an obligate chain terminator [12,13] and, in contrast to PMEG, CDV has been used to manage human papillomavirus (HPV)-induced benign and malignant hyperproliferation with minimal if any side-effects, as described in several case reports and some phase II/III clinical trials [15-20]. Recently, a phase II clinical trial was conducted in Belgium to evaluate the safety and efficacy of CDV in the treatment of high grade cervical lesions (NCT01303328). Full data analysis of this Phase II clinical trial will be provided during the next months.

Cidofovir antitumor properties were also demonstrated in different animal models of tumors related to transforming viruses, including Epstein-Barr virus-associated nasopharyngeal carcinoma [21] and HPV-induced cervical carcinoma [22-24] xenografts in athymic-nude mice, polyomavirus-induced hemangiomas in rats [25] and hemangiosarcoma development in mice [26]. Also, CDV proved effective against cottontail rabbit papillomavirus in the domestic rabbit model [27].

We have recently shown that besides inhibition of tumor growth, intratumoral CDV administration had a beneficial effect on the pathology associated with the growth of cervical carcinoma cells in athymic nude mice as demonstrated by a favorable effect on body weight gain, reduced splenomegaly and lower inflammatory state in animals that received the compound *versus* the placebo-treated group [24]. Furthermore, a whole genome gene expression profiling performed on CDV-treated malignant cells and normal keratinocytes allowed us to identify unique signatures in tumor cells compared to normal keratinocytes pointing to a selective drug effect [28]. Among the functions that were distinctly regulated by CDV in malignant and normal cells, the acute phase response was found exclusively activated in transformed cells but not in normal keratinocytes. In addition, cell cycle regulation and DNA repair by homologous recombination was only activated in normal cells [28].

There are several mechanisms by which cancer cells develop drug-resistance and this is often a multi-factorial process. Understanding the mechanisms leading to development of drug-resistance is crucial for the implementation of therapeutic strategies, for providing insights into the effects of anticancer drugs on specific cellular functions, and also for predicting how acquisition of drug-resistance impacts tumorigenicity and pathogenicity. Therefore, we established, from the cervical carcinoma cell line SiHa (HPV16^+^), a CDV-resistant cell subline (denoted SiHa_*CDV*_) by stepwise dose escalation of CDV. We investigated the *in vitro* and *in vivo* phenotyping and growth rate of SiHa_*CDV*_ compared to parental cells (SiHa_*parental*_). Also, we evaluated the differential gene expression profiles between SiHa_*parental*_ and SiHa_*CDV*_ by microarray analysis in order to identify genes changing expression upon selection of cells for CDV-resistance. In the present study, we focused on the analysis of functions and pathways involved in the inflammatory response that changed in SiHa cells following acquisition of CDV-resistance. Importantly, we also examined whether SiHa cells that acquired resistance to CDV were impaired in pathogenicity in the xenograft model.

## Results

### In vitro phenotyping of SiHa_*CDV*_

**Figure 1:**
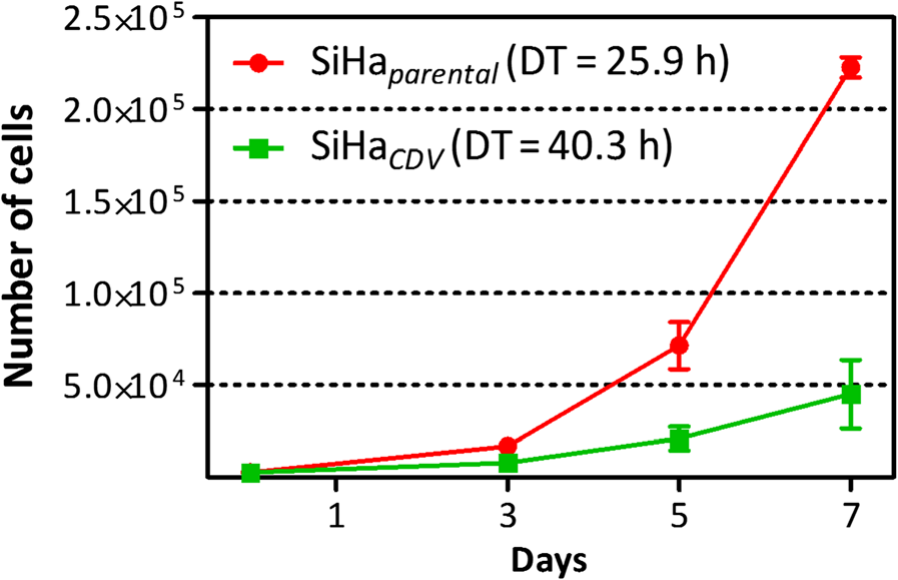
**Growth rate of SiHa**_***CDV ***_**compared to SiHa**_***parental***_**.** Cells were seeded at a density of 2.5 x 10^3^ cells per well in a volume of 0.1 ml into 96-well microtiter plates and allowed to proliferate. At several time-points, cells were trypsinized and the number of cells was determined with a Coulter Counter. Doubling time (DT) was calculated with the formula: DT = (t - t_0_)/(log2N - log2N_0_), where t and t_0_ are the times at which the cells were counted, and N and N_0_ are the cell numbers at times t and t_0_.

SiHa cells (SiHa_*parental*_) were selected for CDV-resistance (SiHa_*CDV*_) following continuous *in vitro* exposure to the drug for approximately 45 passages. The resulting SiHa_*CDV*_ presented a reduced growth rate compared to SiHa_*parental*_ (doubling time of 40 h *versus* 26 h) (Figure 1).

A stable CDV-resistant phenotype in the absence of selective drug pressure was found for the SiHa_*CDV*_. When evaluated in terms of cell growth inhibition (Table 1), a fold-resistance of >100 against CDV was determined after 7 days of incubation with the drug. SiHa_*CDV*_ displayed <10-fold cross-resistance to the cytosine analogue Ara-C and to two unrelated ANPs (i.e. PMEG and cPr-PMEDAP). It should be noted that the antiproliferative effects of CDV (but not of PMEG or cPr-PMEDAP) for SiHa_*parental*_ were time-dependent, pointing to a different mechanism of antiproliferative effects for these drugs, in agreement with our previous report [29].

**Table 1:** **Growth inhibition of SiHa**_***parental***_**and SiHa**_***CDV***_**after incubation with CDV, PMEG, cPr-PMEDAP or Ara-C**

**Compound**	**Cell line**	**CC**_**50**_**(μM)**			**Fold-resistance at day 7**
		**Day 3**	**Day 5**	**Day 7**	
CDV	SiHa_*parental*_	77.1 ± 22.5.1	15.2 ± 13.6	6.3 ± 6.0	>100
	SiHa_*CDV*_	>634.7	>634.7	>634.7	
PMEG	SiHa_*parental*_	0.85 ± 1.14	0.16 ± 0.16	0.10 ± 0.07	2.3
	SiHa_*CDV*_	1.79 ± 2.34	0.39 ± 0.33	0.23 ± 0.016	
cPr-PMEDAP	SiHa_*parental*_	1.22 ± 0.67	0.21 ± 0.15	0.21 ± 0.06	2.9
	SiHa_*CDV*_	3.44 ± 0.52	1.22 ± 0.34	0.61 ± 0.09	
Ara-C	SiHa_*parental*_	0.08 ± 0.04	0.06 ± 0.02	0.13 ± 0.02	8.8
	SiHa_*CDV*_	1.15 ± 0.37	0.99 ± 0.37	1.15 ± 0.49	

Cells were seeded at a density of 2.5 x 10^3^ cells per well in a volume of 0.1 ml into 96-well microtiter plates and allowed to proliferate for 24 h. At this time, media containing different drug concentrations (tested in duplicate) were added (100 μl/well). After 3, 5, and 7 days of incubation, the cells were trypsinized and the number of cells was determined with a Coulter Counter. Antiproliferative effects were expressed as CC_50_ (50% cytostatic concentration), or concentration required to reduce cell growth by 50% (relative to the number of cells in the untreated control cell cultures). Data are mean ± standard deviation of at least two independent experiments. Fold-resistance was calculated as the ratio CC_50_ for SiHa_*CDV*_ to CC_50_ for SiHa_*parental*_.

An inhibition of 93% (SiHa_*parental*_) and 11% (SiHa_*CDV*_) in the number of cells was afforded by CDV treatment at 158.7 μM for 7 days. To compare CDV effects on induction of apoptosis in these cell cultures, annexin V and PI staining was performed. Annexin V stains phosphatidylserine, a negatively charged phospholipid that is translocated from the inner leaflet of the plasma membrane to the outer leaflet during early apoptosis. Since PI does not enter into cells with intact membranes, it was used to identify necrotic cells. SiHa_*parental*_ treated with CDV (at 158.7 μM and 63.5 μM) for 7 days showed increased percentage of apoptotic cells and diminished amounts of viable cells (Table 2). In contrast, SiHa_*CDV*_ were totally refractory to CDV-induced apoptosis while they were still able to respond to PMEG, albeit to a lower extent compared to SiHa_*parental*_. No signs of cell-death by necrosis were seen in any of the two cell lines following treatment with either CDV or PMEG.

**Table 2:** **Apoptosis detection by annexin V binding/PI uptake assay in SiHa**_***parental***_**and SiHa**_***CDV***_

	**SiHa**_***parental***_				**SiHa**_***CDV***_			
	**Untreated**	**CDV 158.7 μM**	**CDV 63.5 μM**	**PMEG 6.5 μM**	**Untreated**	**CDV 158.7 μM**	**CDV 63.5 μM**	**PMEG 6.5 μM**
Early apoptotic	1.2 ± 0.9	22.1 ± 0.6	8.4 ± 1.6	64.2 ± 1.2	4.5 ± 2.0	2.3 ± 0.1	2.4 ± 0.2	30.6 ± 4.3
Late apoptotic	0.6 ± 0.2	5.0 ± 5.6	2.7 ± 2.5	6.0 ± 4.2	0.9 ± 0.4	0.6 ± 0.1	0.6 ± 0.1	3.9 ± 0.7
Viable	97.2 ± 2.2	72.2 ± 6.6	88.0 ± 3.9	29.4 ± 3.0	94.5 ± 2.2	96.3 ± 0.5	96.5 ± 0.3	64.9 ± 1.2
Necrotic	0.4 ± 0.3	0.7 ± 0.3	0.9 ± 0.2	0.4 ± 0.1	0.2 ± 0.1	0.8 ± 0.6	0.5 ± 0.2	0.7 ± 0.3

Cells were seeded in 6-well microtiter plates at a density of 5 × 10^4^ cells/well and allowed to proliferate for 24 h. Culture medium was removed and medium containing the compounds was added. At 7 days post-treatment, cells were harvested with EDTA/PBS (760 mg/L). After rinsing the cells with PBS, the cell pellets were simultaneously stained with annexin-FITC and PI using the annexin-V-FITC staining kit (BD Biosciences, San Jose CA) and analyzed by flow cytometry. Dual fluorescence dot plots were obtained following combined annexin V-FITC and PI staining. Data represent the percentages in each quadrant of the dot plots and are the mean ± SD of at least two stainings. Viable (annexin V^-^/PI^-^) early apoptotic (annexin V^+^/PI^-^), necrotic (annexin V^-^/PI^+^), and late apoptotic (annexin V^+^/PI^+^) cells.

### Differentially expressed genes upon acquisition of CDV-resistance

Gene expression profiling by microarray was performed to identify potential mechanisms associated with CDV-resistance. A total of 1,340 expression changes were identified in SiHa_*CDV*_ compared with SiHa_*parental*_, 777 genes being upregulated and 563 downregulated. To validate the microarray results, transcript levels of four genes (randomly selected before bioinformatics analysis) were evaluated by qPCR, the expression patterns totally matching the microarray data (Additional file 1).

Functional classification of differentially expressed genes showed that they were implicated in a variety of diverse and widely distributed functional categories and biochemical pathways: 12 functional categories and 106 canonical pathways were associated with acquisition of CDV-resistance in SiHa cells (data not shown).

In the present study, we focused on the inflammatory response (one of the 12 functional categories associated with acquisition of CDV-resistance), based on our previous findings showing that CDV treatment of three malignant cells (including SiHa_*parental*_) and primary human keratinocytes allowed the identification of ‘acute phase response signaling’ as a pathway exclusively modulated by CDV in transformed cells but not in normal cells [28]. When analyzing the immune response functional category in SiHa_*CDV*_*versus* SiHa_*parental*_, CDV-resistance was linked to a decrease (negative z-score) in four functional annotations: ‘inflammatory response’, ‘activation of granulocytes’, ‘inflammation of organ’, and ‘activation of neutrophils’ (Additional file 2).

Twenty-one out of 106 canonical pathways affected by the changes in gene expression when comparing SiHa_*CDV*_ and SiHa_*parental*_ were related to immune response (Additional file 3). Several interleukin signaling pathways (such as ‘IL-1 signaling’, ‘IL-6 signaling’, ‘IL-8 signaling’, ‘IL-9 signaling’, ‘IL-10 signaling’) as well as ‘interferon signaling’, the endogenous danger signaling pathway ‘HMGB1 signaling’, the prototypical proinflammatory signaling pathway ‘NF-κB signaling’, the ‘acute phase response signaling’, ‘Toll-like receptor signaling’, and ‘MSP-RON signaling pathway’, were among the several inflammatory response-related pathways altered following acquisition of CDV-resistance in SiHa cells.

**Figure 2:**
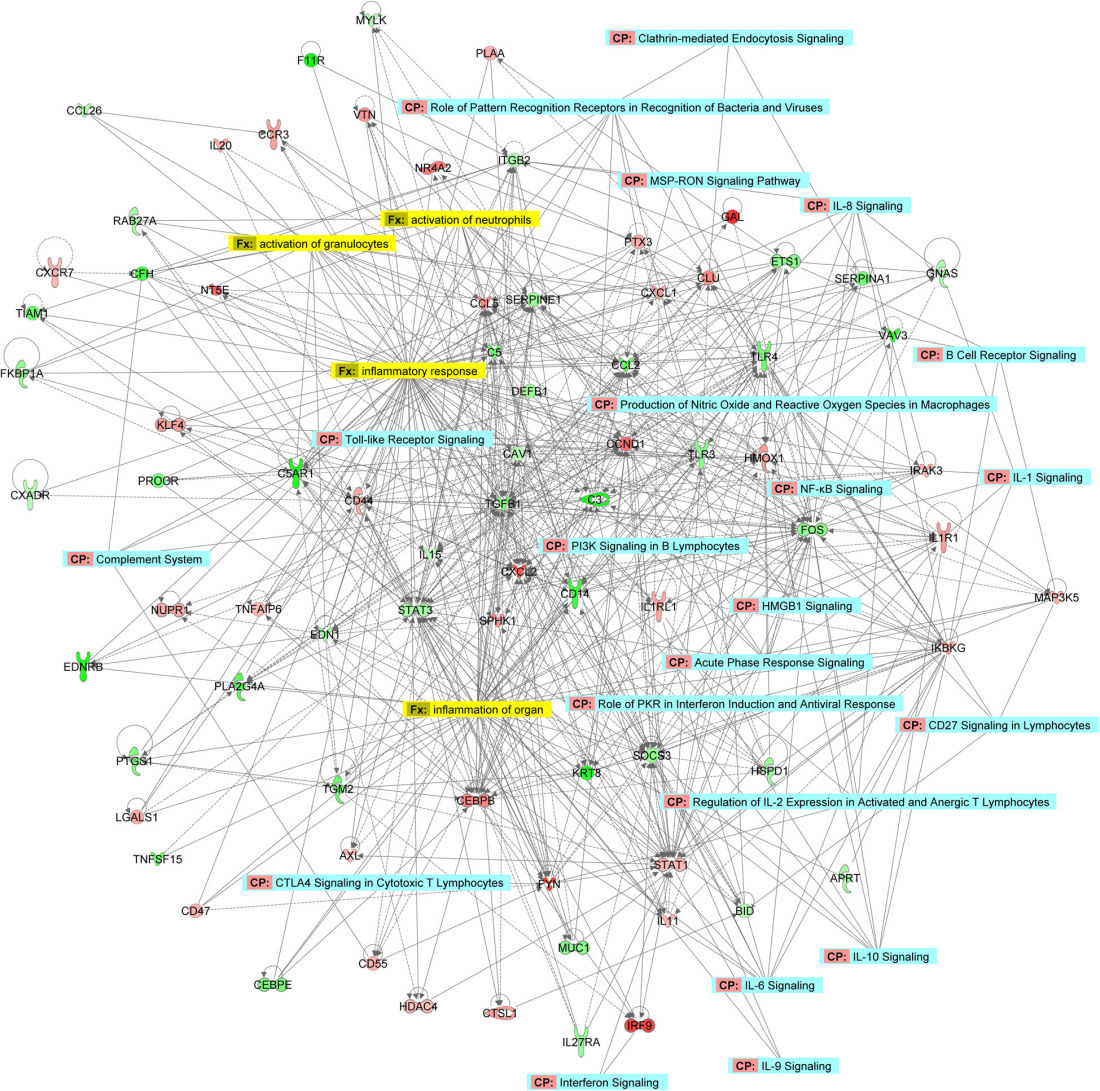
**Network of inflammatory response and corresponding transcripts.** Networks were constructed with IPA software using genes DE involved in ‘inflammatory response’. A network is a graphical representation of the molecular relationships between molecules (nodes). The biological relationship between two nodes is represented as an edge (line) connecting two nodes. All edges are supported by information from the literature stored in the Ingenuity Pathways Knowledge Base. A solid line represents a direct interaction between two gene products and a dotted line means there is an indirect interaction. The intensity of the node color indicates the degree of up-regulation (red) or down-regulation (green) when comparing SiHa_*CDV *_*versus* SiHa_*parental*_ cells. Canonical pathways identified by IPA in the networks are shown in blue while the functional annotations are shown in yellow.

Using stringent criteria for microarray analysis (≥ 2-fold change in expression and p-value < 0.05), 173 genes related to the ‘inflammatory response’ function were identified as differentially expressed when comparing SiHa_*CDV*_ and SiHa_*parental*_, 86 being upregulated and 87 downregulated (Additional file 4). In order to visualize the interactions of differentially expressed genes involved in the inflammatory response, a network was constructed based on the differentially expressed genes involved in the inflammatory response function (Figure 2). Gene networks represent intermolecular connections among interacting genes based on functional knowledge inputs stored in the IPKB (ingenuity Pathways Knowledge Base). The different pathways and functions in the inflammatory response associated with CDV-resistance are indicated in this network. Among several genes changing expression upon acquisition of CDV-resistance, decreased gene expression of *TGFB1* (transforming growth factor beta-1), *STAT3* [signal transducer and activator of transcription 3 (acute-phase response factor)], *SOCS3* (suppressor of cytokine signaling 3), *FOS* (proto-oncogene c-Fos or activator protein 1), *TLR3* and *TLR4* (toll-like receptor 3 and 4) and increased gene expression of *CCND1* (cyclin D), *CXCL2* [chemokine (C-X-C motif) ligand 1], *CEBPB* (interleukin 6-dependent DNA-binding protein) and *STAT1* (signal transducer and activator of transcription-1) appeared to play a central role in the changes in the inflammatory response that accompanied the development of CDV-resistance (Figure 2). These genes were implicated in several pathways and functions changed in SiHa_*CDV*_*versus* SiHa_*parental*_.

### In vivo phenotyping, tumorigenicity and pathogenicity of SiHa_*CDV*_

In a following step, we examined whether SiHa_*CDV*_ presented a resistant phenotype *in vivo*. Four weeks intratumoral treatment with CDV of athymic nude mice bearing the SiHa_*CDV*_ xenografts resulted in a moderate but not significant effect on tumor growth (Additional file 5). In contrast, the same treatment given to mice harboring the SiHa_*parental*_ xenograft caused a remarkable and significant suppression of tumor growth, in agreement with our previous report [24]. Interestingly, tumor size was significantly lower in the SiHa_*CDV*_ cohort than in the SiHa_*parental*_ group.

**Figure 3:**
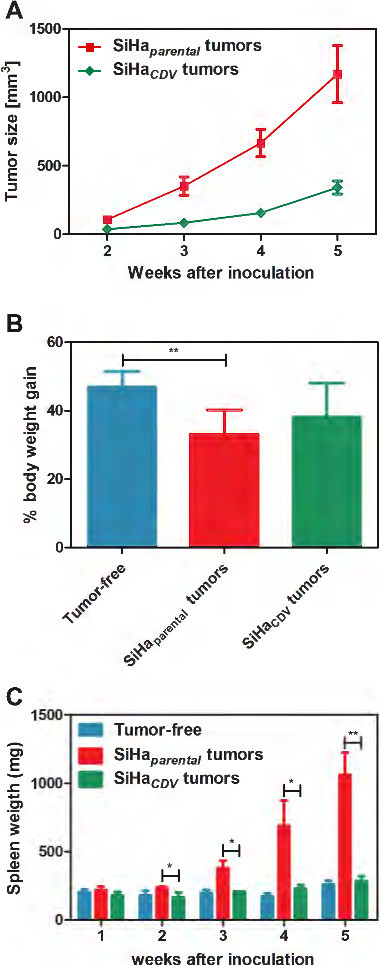
**Growth of SiHa**_***CDV ***_**compared to SiHa**_***parental ***_**in a xenograft model in athymic nude mice. (A)** Tumor growth was determined in *nu/nu* mice bearing SiHa_*CDV*_ or SiHa_*parental*_ grown as xenograft at different time points post-inoculation of the cells. Data were collected from two independent experiments (including a total of 15 mice/group) over a period of 5 weeks and are presented as the average tumor volume (mm^3^) ± SEM. Tumors were measured by means of a digital caliper in two directions (perpendicular diameters) and the formula V = (4πab^2^)/3, with ‘a’ and ‘b’ being the largest and smallest radius of the tumor, respectively, was applied to calculate the tumor volume. **(B)** Body weight of the mice (excluding the tumor mass) was determined after 5 weeks of inoculation of the cells and body weight gain was calculated as percentage of the initial body weight of the mice at the start of the experiment. Data shown represent the average (±SD) of five individual mice per group. Healthy control mice gained 47.0 ± 4.4% of their initial body weight, while mice bearing the SiHa_*parental*_ cells only gained 33.2 ± 7.1% (p < 0.01 compared to tumor-free mice). An intermediate body weight gain of 38.2 ± 9.8 (p > 0.05) compared to healthy control mice was recorded for mice with SiHa_*CDV*_. **(C)** Weight of the spleens was assessed at different weeks post-inoculation of the cells.

Therefore, we investigated the kinetics of growth and the pathogenicity of SiHa_*CDV*_ in the xenograft model in athymic nude mice. The growth rate of SiHa_*CDV*_ tumors was significantly reduced compared to SiHa_*parental*_ (Figure 3A). SiHa_*parental*_ tumor size (351.6 ± 259.8 mm^3^) at week 3 was equivalent to that of SiHa_*CDV*_ (342.0 ± 182.3 mm^3^) at week 5 (Figure 3A). In contrast to animals inoculated with SiHa_*parental*_, the cohort bearing SiHa_*CDV*_ tumors did not develop signs of wasting syndrome. Body weight gain determined after subtraction of the tumor weight showed that the SiHa_*parental*_ group gained significant less weight than tumor-free control animals (Figure 3B). Despite no statistically significant differences, mice with SiHa_*CDV*_ xenografts presented an intermediate body weight gain.

Blood test analysis at week 5 showed similar results in the SiHa_*CDV*_ tumor and tumor-free groups, except for a significant increase in the platelet count in mice bearing SiHa_*CDV*_ tumors (Table 3). By contrast, the SiHa_*parental*_ group showed significant changes in several blood parameters compared to either tumor-free or SiHa_*CDV*_ tumor mice. Hence, animals harboring SiHa_*parental*_ tumors showed markedly higher white blood cell (WBC) count and lower RBC (red blood cell) count than tumor-free animals, most likely as a consequence of the growth of the xenograft and the subsequent induced inflammation. The increase in the platelet number observed in the SiHa_*parental*_ group (but not in the SiHa_*CDV*_ cohort) was accompanied by an increase in the mean platelet volume. The growth of SiHa_*parental*_ in mice also caused a marked decrease in hemoglobin concentration and in the mean corpuscular hemoglobin concentration, pointing to anemia. Also, the liver enzymes aspartate aminotransferase and gamma-glutamyl transferase as well as lactate dehydrogenase were increased in the blood of the SiHa_*parental*_ cohort. Elevation of creatine kinase (which is an indication of damage to muscle) and of creatinine (a sign of impaired kidney function) was also seen in the SiHa_*parental*_ group. Taken together, these data clearly signified a pronounced alteration of hematological and chemical blood parameters in the SiHa_*parental*_ group and a lesser pathogenic effect of SiHa_*CDV*_*versus* SiHa_*parental*_ in the xenograft model.

**Table 3:** Complete blood count of tumor-bearing mice

**Blood test**	**Average ± SD**			***p*-value (*t*-test)**		
	**Tumor-free**	**SiHa**_***parental ***_**tumor**	**SiHa**_***CDV ***_**tumor**	**Tumor-free *vs *** **SiHa**_***parental***_	**Tumor-free *vs *** **SiHa**_***CDV***_	**SiHa**_***parental ***_ ***vs *** **SiHa**_**CDV**_
**Hematology**						
Hemoglobin [g/dl]	13.9 ± 0.3	10.6 ± 1.7	12.9 ± 1.2	*	n.s.	n.s.
Hematocrit	0.46 ± 0.01	0.37 ± 0.06	0.43 ± 0.03	n.s.	n.s.	n.s.
RBC count [10^12^/l]	8.91 ± 0.32	7.08 ± 1.17	8.38 ± 0.91	*	n.s.	n.s.
MCV [fl]	51.2 ± 1.5	52.9 ± 0.5	51.7 ± 1.9	n.s.	n.s.	n.s.
MCH [pg]	15.6 ± 0.5	≤ 15	15.9 ± 0.2	-	n.s.	-
MCHC [g/dl]	30.6 ± 0.7	28.2 ± 0.5	29.8 ± 0.7	**	n.s.	*
RDW [%]	17.4 ± 0.8	18.4 ± 0.8	18.2 ± 1.0	n.s.	n.s.	n.s.
Reticulocyte count [%]	3.1 ± 0.8	2.6 ± 1.6	3.3 ± 0.5	n.s.	n.s.	n.s.
IRF [%]	41.0 ± 10.3	24.8 ± 10.3	36.7 ± 5.4	n.s.	n.s.	n.s.
Platelet count [10^9^/l]	1037 ± 113	1566 ± 22	1783 ± 240	**	**	n.s.
MPV [fl]	7.1 ± 0.2	7.8 ± 0.4	7.2 ± 0.1	*	n.s.	*
WBC count [10^9^/l]	2.17 ± 0.22	208.11 ± 107.15	9.39 ± 6.63	*	n.s.	**
**Chemistry**						
Creatinine [mg/dl]	< 0.20	< 0.20	< 0.20	-	-	-
AST [U/l]	88 ± 13	392 ± 188	127 ± 39	*	n.s.	*
ALT [U/l]	48 ± 11	63 ± 46	33 ± 16	n.s.	n.s.	n.s.
GGT [U/l]	< 3	12 ± 6	< 3	-	-	-
Bilirubin [mg/dl]	< 10	< 10	< 10	-	-	-
CK [U/l]	144 ± 16	400 ± 60	224 ± 124	***	n.s.	n.s.
LDH [U/l]	1263 ± 361	8966 ± 1613	3253 ± 1772	**	n.s.	*

Five weeks after inoculation of the tumor cells, blood was collected and examined by complete blood count. RBC: red blood cell; MCV: mean cell volume; MCH: mean corpuscular hemoglobin; MCHC: mean corpuscular hemoglobin concentration; RDW: red blood cell distribution width; IRF: immature reticulocyte count; MPV: mean platelet volume; WBC: white blood cell; AST: aspartate aminotransferase; ALT: alanine aminotransferase; GGT: gamma-glutamyl transferase; CK: creatine kinase; LDH: lactate dehydrogenase. Statistical significance was assessed based on unpaired two-tailed Student’s t-test, n.s.: not significant; *: significant (p values < 0.05); **: very significant (p < 0.01); and ***: extremely significant (p < 0.001).

**Figure 4:**
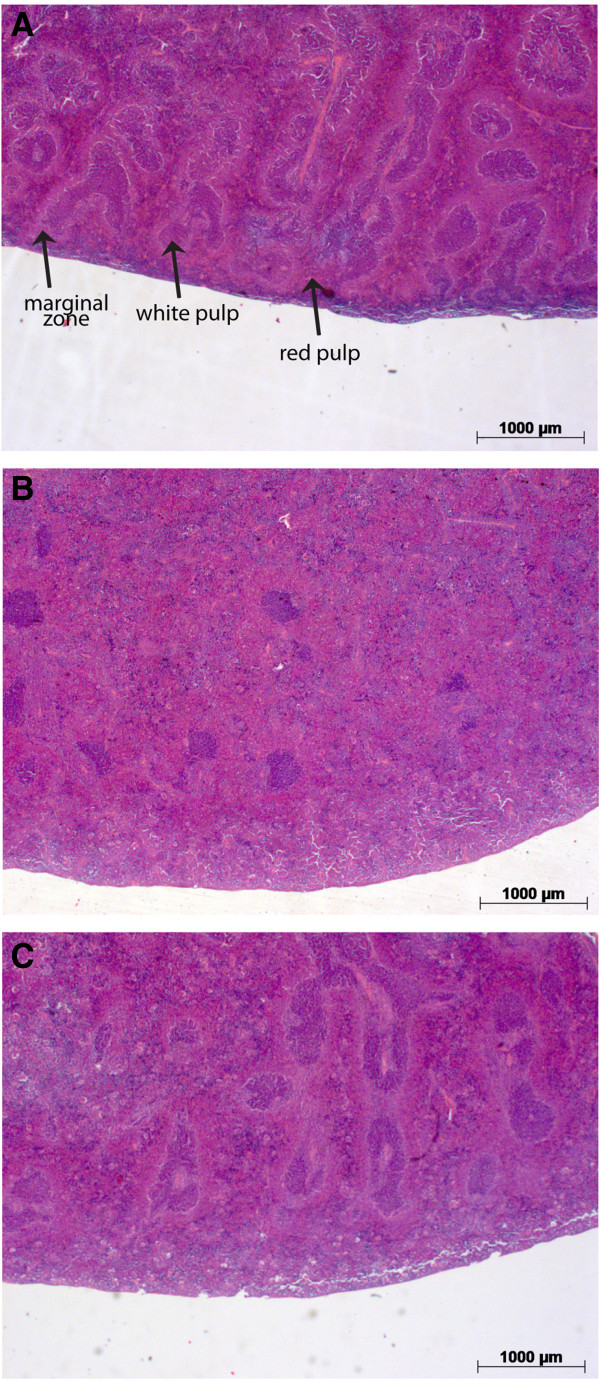
**Spleen pathology of mice inoculated with SiHa**_***CDV ***_***versus *SiHa**_***parental***_**.** Tissues were fixed in neutral buffered formalin, subsequently embedded in paraffin and 5 μm sections were hematoxylin-eosin (H & E) stained and microscopically examined. Representative H&E stained tissue sections of spleens from tumor-free **(A)**, and mice bearing SiHa_*parental*_**(B)** or SiHa_*CDV*_**(C)** xenografts, five weeks after inoculation of the cells (2.5x magnification). Splenomegaly caused by SiHa_*parental*_ tumors was associated with severe changes in the relative size of the red and white pulp with an expanded red pulp, only sparse spots of white pulp and virtually no marginal zones remaining. In animals inoculated with SiHa_*CDV*_, no major alterations in the morphology of the spleen were noted.

The SiHa_*parental*_ cohort showed a time-dependent splenomegaly that started at week 3, while the group inoculated with SiHa_*CDV*_ had no signs of splenomegaly at any time point (Figure 3C). Splenomegaly caused by SiHa_*parental*_ tumors was associated with severe changes in the relative size of the red and white pulp and with infiltration of polymorphonuclear leukocytes in the extended red pulp (Figure 4). In the SiHa_*CDV*_ cohort, no considerable alterations in the morphology of the spleen were noted.

**Figure 5:**
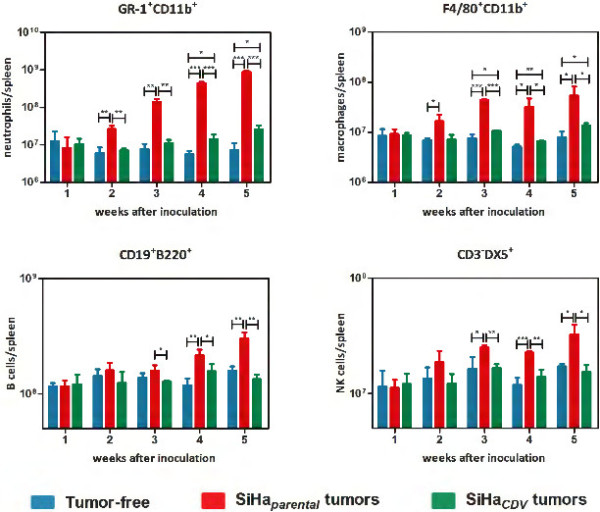
**Flow cytometric analysis of immune cell populations in the spleen of mice bearing SiHa**_***CDV ***_**or SiHa**_***parental ***_**xenografts compared to healthy animals.** Gr1^+^CD11b^+^ neutrophils, F4/80^+^CD11b^+^ macrophages, CD19^+^B220^+^ B-cells and CD3^-^DX5^+^ NK-cells are depicted as number of cells per spleen. Each bar represents the average (±SD) of three individual mice.

When the different immune cell types in the spleens were quantified, striking differences were noted between animals inoculated with the two SiHa cell lines. Hence, the SiHa_*parental*_ group had a very pronounced and time-dependent increase in the number of neutrophils per spleen compared to healthy control mice (Figure 5). This increase could be attributed not only to splenomegaly but also to a higher percentage of neutrophils within the splenocytes in SiHa_*parental*_ mice compared to healthy animals (data not shown). Splenomegaly in mice inoculated with SiHa_*parental*_ was also associated with increased numbers of macrophages, NK-cells and B-cells per spleen compared to healthy animals.

When comparing the SiHa_*CDV*_ group with healthy animals, a slight increase (although significant) in the amounts of macrophages and neutrophils was measured, respectively, from weeks 3 and 4 onwards, while the numbers of NK-cells and B-cells did not differ between the two cohorts throughout the entire experiment (Figure 5).

The SiHa_*CDV*_ cohort had a much lower amount of neutrophils per spleen compared to the SiHa_*parental*_ group which became evident from 2 weeks onwards. The number of macrophages, NK cells and B cells were lower in animals harboring SiHa_*CDV*_ xenografts, starting from week 3 onwards, compared to the SiHa_*parental*_ cohort.

**Figure 6:**
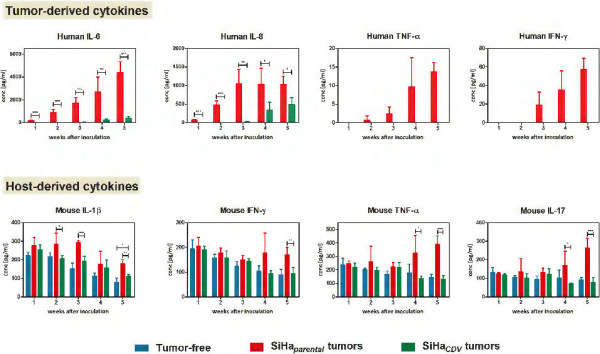
**Host and human cytokine levels in the sera of mice harboring SiHa**_***CDV ***_**or SiHa**_***parental ***_**tumors compared to tumor-free animals.** Cytokine levels in the sera of mice were determined after 1–5 weeks post-inoculation of the cells. Each bar represents the average (±SD) of three individual mice. Human cytokines GM-CSF, IL-1b, IL-2, IL-4, IL-5, IL-7, IL-10, IL-12, IL-13, IL-17, MIP1β, M-CSF, TNF-β were undetectable. The mouse cytokines IL-2, IL-4, IL-5, IL-10, granulocyte-macrophage colony-stimulating factor (GM-CSF), IL-6, IL-13, macrophage colony-stimulating factor (M-CSF) and IL-18 were detectable but no differences among the three groups of mice were observed.

The groups with SiHa_*parental*_ and SiHa_*CDV*_ tumors also differed in their ability to modulate tumor-derived cytokines and host-derived cytokines in the sera of mice (Figure 6). Most of the human-derived cytokines measured in the sera of tumor-bearing mice were undetectable. However, four tumor-derived cytokines (IL-6, IL-8, TNF-α, and IFN-γ) were found time-dependently induced in the SiHa_*parental*_ group. Human TNF-α and INF-γ were undetectable at any time point in the SiHa_*CDV*_ group while human IL-6 and human IL-8 were detected at very low levels starting at week 3. Notably, SiHa_*parental*_ cells were able to produce very high levels of IL-6. The decreased production of IL-6 by SiHa_*CDV*_ compared to SiHa_*parental*_ was confirmed by an ELISA assay performed with cell-culture supernatants from both cell types (Additional file 6).

Figure 6 also shows the mouse-derived cytokines (IL-1β, IFN-γ, TNF-α, and IL-17) differently detected in tumor-bearing mice *versus* control healthy animals. In the tumor-free group, the host derived cytokines IL-1β, IFN-γ and TNF-α showed a time-dependent decline in concentration as a consequence of immune cell maturation while IL-17 levels remained stable in function of time. Sera of older mice showed lower and stable concentrations of cytokines (data not shown), pointing to an effect of immune maturation on cytokine levels rather than an effect of housing or experimental conditions. Differences between healthy mice and the SiHa_*parental*_ cohort started to be noticeable from week 2 (IL-1β), 4 (TNF-α and IL-17) or 5 (IFN-γ). While IFN-γ, TNF-α and IL-17 levels remained stable or decreased during the first weeks in the SiHa_*parental*_ group, their levels raised from week 4 onwards. Although IL-1β still decreased in function of time in mice with SiHa_*parental*_ tumors, the levels were significantly higher than in healthy animals. Except for IL-1β at week 5, host-derived cytokines levels did not significantly differ between the tumor-free and the SiHa_*CDV*_ cohorts at any time point post-inoculation of the cells, pointing to a markedly diminished host inflammatory response compared to SiHa_*parental*_ xenografts.

## Discussion

In the present study, we showed that SiHa cells that acquired CDV-resistance proved to be refractory to CDV-antiproliferative effects and to CDV-induced apoptosis *in vitro*. These HPV-16 positive cervical carcinoma cells demonstrated a high barrier for the development of resistance to CDV as selection required prolonged exposure to CDV (approximately 45 passages during a 2-years time).

Genome wide gene expression analysis has been previously used to identify gene expression signatures associated with resistance to chemotherapeutic agents [30-32]. Here, we compared microarray gene expression values of SiHa_*CDV*_ with SiHa_*parental*_ and bioinformatics analysis revealed the implication of a variety of biological functions and pathways (linked to cell death, cell growth and proliferation, cellular movement, metabolism, cell and tissue development as well as inflammatory response) changing following acquisition of resistance to CDV. Thus, it appears that acquisition of CDV-resistance is a multifactorial process, which is in agreement with findings on development of resistance to several chemotherapeutics [33].

By examining the identities of the genes in the ‘inflammatory response’ exhibiting changes in expression upon acquisition of CDV-resistance, it can be assumed that the identified genes may not be the ‘drivers’ of drug-resistance, but they changed expression as a consequence of altered expression of the ‘driver’ genes. Candidate genes that should be further explored include c-Fos, c-Jun, PI3K and MAPK since they were changing expression upon acquisition of CDV-resistance and were involved in most of the inflammatory response pathways. Further investigations to elucidate the genes that drive acquisition of CDV-resistance are currently ongoing.

The changes in inflammatory response observed in cells that acquire CDV-resistance are expected to be a consequence of the development of CDV-resistance rather than the cause of the resistant phenotype *in vitro*. While not causing the resistant phenotype per se, the alterations in inflammatory response are expected to affect the tumor microenvironment *in vivo* and to contribute to the observed reduction in pathogenicity and tumorigenicity. Therefore, it was interesting to investigate how acquisition of CDV-resistance in SiHa cells affected the inflammatory response induced by these cells in an athymic nude mice xenograft model.

In the *in vitro* setting, SiHa_*CDV*_ proved clearly resistant to CDV but this must occur via a mechanism that does not directly involve cells of the immune system or the tumor microenvironment. In contrast, *in vivo*, the decreased inflammatory response observed with SiHa_*CDV*_ compared to SiHa_*parental*_ affected the tumor microenvironment and contributed to a reduced pathogenicity of the xenografts as SiHa_*CDV*_ provoked less inflammation in the xenograft model (evidenced by a reduced production of mice- and human-derived cytokines, diminished effect on chemical and hematological blood parameters, lower number of immune cells in the spleen, and lesser splenomegaly compared to parental cells).

In contrast to SiHa_*CDV*_, SiHa_*parental*_ generated a pronounced stimulation of immune cells (mostly neutrophils) when evaluated in comparison to healthy animals. One could argue that the reduced induction of neutrophils, macrophages, B-cells and NK-cells by SiHa_*CDV*_ could be the consequence of reduced growth rate observed for the SiHa_*CDV*_ not only *in vitro* but also *in vivo*. Yet, SiHa_*parental*_ tumor size at week 3 was equivalent to that of SiHa_*CDV*_ at week 5 (tumor size of, respectively, 351.6 ± 259.8 mm^3^ and 342.0 ± 182.3 mm^3^) while the amount of neutrophils, macrophages and NK-cells was considerable higher in mice with SiHa_*parental*_ xenografts than in those with SiHa_*CDV*_ tumors at these time points. Similarly, when putting side by side the SiHa_*parental*_ and SiHa_*CDV*_ groups at the moment that they have an equivalent tumor size (week 3 and week 5, respectively), IL-1β was detected in higher amounts in the SiHa_*parental*_ cohort. IL-1β plays a key role in the regulation of neutrophil recruitment through up-regulation of endothelial adhesion molecule expression on endothelium and through induction of local chemokine production (including IL-8) production [34], and indeed lower IL-1β levels correlated with lower numbers of neutrophils in the SiHa_*CDV*_ cohort.

Neutrophils and macrophages have a major role in defense mechanisms and protect the host from injury and infections. However, they were shown to infiltrate most solid cancers and tumor-associated macrophages (TAMs) and tumor-associated neutrophils (TANs) were shown to be involved in stimulation of tumor growth, their densities being linked to poor outcomes and shorter survival in several cancer types [35,36]. A recent study showed that elevated white blood cells and neutrophil counts at the time of recurrence diagnosis correlated with shorter survival in patients with recurrent cervical cancer [37]. In other cancers, such as colon cancer, small cell lung carcinoma, and melanoma, an elevated neutrophil-to-lymphocyte ratio also predicted a significantly higher risk of death [38-40].

Recently, a role for the spleen as a site for storage and rapid deployment of monocytes to inflammatory sites has been unraveled, identifying splenic monocytes as a resource that the body uses to regulate inflammation [41]. Cortez-Retamoza and colleagues [42] demonstrated the function of the spleen as a reservoir of monocytes using a mouse model of lung adenocarcinoma. High numbers of TAMs and TANs relocated from the spleen to the tumor stroma. Furthermore, removal of the spleen (either before or after tumor initiation) reduced TAMs and TANs responses markedly and delayed tumor growth [42]. Local accumulation of granulocytes and macrophage progenitors in the splenic red pulp was linked to the reservoir capacity of the spleen during tumor progression. Our data showing an infiltration of polymorphonuclear leukocytes in the extended red pulp in the SiHa_*parental*_ xenograft cohort (but not in the SiHa_*CDV*_ one) suggest that the spleen might also play an important role as reservoir of monocytes. Moreover, a pronounced increase in the number of WBC was detected in the SiHa_*parental*_ but not in the SiHa_*CDV*_ group. Our microarray data also indicated that acquisition of CDV-resistance was associated with reduction of ‘inflammatory response’, ‘activation of granulocytes’, inflammation of organ’ and ‘activation of neutrophils’ (Additional file 2), which can explain the diminished stimulation of the production of neutrophils and macrophages by the host. Decreased expression of genes whose products are responsible for activation of neutrophils and/or granulocytes (such as complement components, endothelin 1, IL-15, integrin β2, monocyte chemoattractant protein 1, macrophage inflammatory protein 4α, protein kinase C inhibitor 2, GTP-binding protein Ram, and monocyte differentiation antigen CD14) point to a decreased capacity of SiHa_*CDV*_ cells to activate and attract neutrophils and macrophages at the tumor site compared to SiHa_*parental*_.

Overall, our data showed that SiHa_*CDV*_ elicited a reduced inflammatory response in the xenograft model when evaluated in comparison with SiHa_*parental*_. Inflammation is present in almost all cancer tissues and the inflammatory state is necessary in tumor tissue remodeling, angiogenesis and metastasis [43-45]. Altered expression of cytokines and growth factors is crucial in the malignant transformation of many cancers. Inflammation, actually ‘smoldering’ inflammation, is now considered as one of the hallmarks of cancer [43,46]. Recent studies pointed out the importance of cytokine profiles in patients with cervical intraepithelial and invasive neoplasia, suggesting that tumor progression is dependent on suppression of cellular immunity [47,48]. Hence, decreased levels of Th1 cytokines were reported in high-grade lesions, consistent with the role of Th1 cytokines as potent activators of cell-mediated immunity [48-50]. Scott and colleagues also demonstrated that persistence of an HPV infection is linked to a failure to express Th1 cytokines [51]. Chronic Th2 type inflammation is commonly seen during persistent infection with high-risk HPV types promoting tumor progression [52]. Furthermore, high-risk HPV types are able to initiate a local Th2 inflammation at an early stage, creating an immunosuppressive microenvironment that contributes to tumor progression [47].

We have previously shown that the production of a number of cytokines by SiHa_*parental*_, including the pro-inflammatory cytokines IL-6, IL-8, TNF-α and IFN-γ, is decreased following CDV therapy in the xenograft model in *nu/nu* mice [24]. Here, we demonstrated that SiHa_*CDV*_ produced significant lower levels of these pro-inflammatory cytokines in mice. These findings were supported by bioinformatics analysis of microarray gene expression profiling that showed alteration of interleukin (IL-1, IL-6, IL-8, IL-9, IL-10) and interferon signaling pathways.

Acquisition of CDV-resistance resulted in inhibition of the IL-6, IL-9, and IL-10 signaling pathways as inferred by a decreased expression of STAT3, SOCS2 and SOCS3. The STAT3 protein is activated through phosphorylation in response to various cytokines and growth factors including IFNs, EGF, IL-5, and IL-6, mediating the expression of a variety of genes in response to cell stimuli, and thus playing a key role in many cellular processes [53,54]. SOCS family members are cytokine-inducible negative regulators of cytokine receptor signaling via the Janus kinase/signal transducer and activation of transcription pathway (the JAK/STAT pathway) [55]. Transcripts encoding SOCS are upregulated in response to cytokine stimulation, and the corresponding SOCS proteins inhibit cytokine-induced signaling pathways. Therefore, SOCS proteins form part of a classical negative feedback circuit [56,57]. Expression of SOCS2 can be induced by a subset of cytokines such as GM-CSF, IL-10 and IFN-γ while that of SOCS3 by IL-6, IL-10 and IFN-γ. It can be inferred that reduced expression of STAT3 and SOCS genes in SiHa_*CDV*_*versus* SiHa_*parental*_ is the consequence of reduced levels of cytokines, and indeed, SiHa_*CDV*_ produced lower levels of pro-inflammatory cytokines (IL-6, IL-8, TNF-α and IFN-γ) in mice.

In the xenograft model, human IL-6, IL-8, and TNF-α are expected to have an important role in the mice pathology because they are known to be biologically active in mice, in contrast to IFN-γ and its receptor that are species specific [58]. SiHa_*parental*_, but not CDV-resistant cells, produced high levels of IL-6. This cytokine is known to induce extensive extramedullar hematopoiesis leading to production of neutrophils that localize to the tumor microenvironment promoting tumor growth by protease-induced angiogenesis [59].

TNF, originally identified for its ability to induce rapid hemorrhagic necrosis of experimental tumors, is now recognized as a central mediator of inflammation, representing one of the molecular links between chronic inflammation and the subsequent development of malignant disease [60]. TNF-α is a strong activator of NF-κB, an injury transcription factor that contributes to cell survival, proliferation, invasion, inflammation and angiogenesis [61]. Tumor promotion by TNF-α can involve diverse pathways, including enhancement of tumor growth and invasion, leukocyte recruitment, angiogenesis and facilitation of mesenchymal transition [62]. SiHa_*CDV*_ showed increased expression of the TNF receptor TNFRSF11B and diminished expression of the TNF ligand TNFSF15 (Additional file 4), which is expected to affect NF-κB activation and apoptosis induction. This hypothesis is based on the fact that TNFRSF11B is a decoy receptor for RANKL (receptor activator of NF-κB ligand) and TRAIL (TNF-related apoptosis-inducing ligand), and that TNFSF15 (which is inducible by TNF and IL-1α) binds to TNFRSF21 (an activator of NF-κB and of apoptosis). Further evidence for an effect on NF-κB activation in SiHa_*CDV*_*versus* SiHa_*parental*_ is provided by increased expression of the TNF associated factor TRAF3 (a known inhibitor of NF-κB activation) and of IKBKG [the regulatory subunit of the inhibitor of kappa B kinase (IKK) complex, also known as NEMO].

The decreased expression of several genes implicated in the HMGB1 (high mobility group box 1) signaling pathways in SiHa_*CDV*_*versus* SiHa_*parental*_ further supports the reduced tumorigenicity and inflammation of cells that acquired CDV-resistance. As post-translational modifications determine intracellular distribution and key functions of HMGB1, changes at the mRNA level for HMGB1 were not detected. However, in the HMGB1 signaling pathway, expression of mitogen-activated protein kinases (MAPKs) and of the serine/threonine kinase AKT3 was reduced in SiHa_*CDV*_*versus* SiHa_*parental*_, leading, respectively, to diminished expression of c-Fos and c-Jun and to regulation of NK-κB. c-Fos and c-Jun form the transcription factor complex AP-1 which regulates gene expression in response to a variety of stimuli (such as cytokines, growth factors, stress, and microbial infections) and controls a number of cellular processes. HMGB1, considered as a prototypic damage-associated molecular pattern (DAMP) molecule, acts as both a ligand and a sensor of the signal-transducing innate responses. Therefore, it can be assumed that a decrease in HMGB1 signaling following acquisition of CDV-resistance may result in lower stimulation of pro-inflammatory cytokines.

Another interesting finding when comparing SiHa_*CDV*_ and SiHa_*parental*_ is their differences in TLR signaling, with TLR3 and TLR4 is downregulated in SiHa_*CDV*_. TLRs activate several signaling elements that results in activation of pro-inflammatory cytokines, regulating apoptosis, antimicrobial response and immune responses. Expression of TLRs in tumor cells can promote inflammation and cell survival in the tumor microenvironment [63,64]. Moreover, expression of TLRs in esophageal squamous carcinoma [65] and in cervical lesions [66] was shown to correlate with disease severity. As TLRs promote tumor cell growth and cytokine secretion, leading to the escape of tumor cells from immune surveillance, it can be assumed that reduced TLR expression in SiHa_*CDV*_ will contribute to a reduced inflammatory response and decreased tumor growth compared to the parental cells.

Further evidence for lower tumorigenicity induced by SiHa_*CDV*_*versus* SiHa_*parental*_ in mice is provided by changes in the ‘MSP/RON signaling pathway’. Macrophage-stimulating protein (MSP) activates the RON receptor tyrosine kinase, which regulates several activities of epithelial cells [67]. The MSP-RON pathway plays also a role in epithelial carcinogenesis and RON is found over-expressed in many breast, colon, and pancreatic tumors [67]. As activation of the MSP-RON pathway directs invasive growth (characterized by increased cell replication, migration, and matrix invasion) [68,69], it can be inferred that the decreased expression of genes involved in this pathway [such as TLR4, TLR3, monocyte chemoattractant protein 1 (CCL2) and integrin β2] in SiHa_*CDV*_*versus* SiHa_*parental*_ will be translated in a reduced tumorigenicity *in vivo*.

In the context of developing CDV as an anti-cancer drug, our findings have therapeutic/biological significance since we showed that acquisition of CDV-resistance is expected to result in a reduced malignant phenotype.

Today, no evidence for the development of resistance to CDV in the treatment of HPV-associated (malignant) lesions has been reported.

## Conclusions

Although several studies have characterized CDV-resistant herpes- [70], and poxviruses [71], this is the first study reporting the *in vivo* characterization of tumor cells selected for CDV-resistance. Similarly to a reduced pathogenicity described for CDV-resistant viruses, development of resistance to CDV as an anti-cancer drug was associated with a marked reduction in pathogenicity. The present study contributes to our understanding on how the alterations in inflammatory response following acquisition of CDV-resistance while not causing the resistant phenotype *per se* affect the tumor microenvironment *in vivo* and contribute to a reduced pathogenicity and tumorigenicity.

## Methods

### Compounds

Cidofovir [CDV, (*S*)-HPMPC, (*S*)-1-(3-hydroxy-2-phosphonylmethoxypropyl)cytosine], PMEG {[9-(2-phosphonylmethoxyethyl)guanine]} and cPr-PMEDAP {cyclo-propyl-9-[2-(phosphonomethoxyethyl]diaminopurine]} were kindly provided by Gilead Sciences, Inc., Foster City, California. Cytarabine [Ara-C, (β-D-Arabinofuranosyl)cytosine] was obtained from Sigma.

### Cells

SiHa cells, HPV16-positive cervical carcinoma (ATCC, # HTB-35™), were maintained in Dulbecco’s modified Eagle’s medium supplemented with 10% fetal calf serum. SiHa cells resistant to CDV were selected by passing the cells under increasing drug concentration for approximately 45 passages (initial drug concentration of 1.6 μM and final concentration of 317.3 μM) during a 2-years time. The parental SiHa cells and those selected for resistance to CDV were denoted SiHa_*parental*_ and SiHa_*CDV*_, respectively. In order to demonstrate that both cell lines were related, short tandem repeat (STR) analysis was performed at the Forensic Laboratory of UZ Leuven (Leuven, Belgium). Despite some small alterations following long-term culturing of the cells, the STR analysis confirmed that SiHa_*parental*_ and SiHa_*CDV*_ were related and thus that the resistant cell line is indeed a derivative of the parental cell line (see Additional file 7).

### Drug-antiproliferative effects and in vitro growth rate

Inhibition of SiHa_*parental*_ and SiHa_*CDV*_ growth was determined following different times of incubation with the compounds. Compounds were tested at different concentrations in a range of 0.63 μM – 634.7 μM for CDV, 0.0065 μM – 6.54 μM for PMEG, 0.061 μM – 60.99 μM for cPr-PMEDAP, and 0.0205 μM – 20.53 μM for Ara-C. Antiproliferative effects were expressed as CC_50_ (50% cystostatic concentration), or concentration required to reduce cell growth by 50%.

Doubling time (DT) of SiHa_*parental*_ and SiHa_*CDV*_ was determined in 48-well microtiter plates from growth curves performed in absence of the drug by using the formula: DT = (t - t_0_)/(log_2_N – log_2_N_0_), where t and t_0_ are the times at which the cells were counted, and N and N_0_ are the cell numbers at times t and t_0_.

### Detection of apoptosis

To differentiate between living, apoptotic and necrotic cells, SiHa_*parental*_ and SiHa_*CDV*_ were grown for 7 days in the presence of CDV or PMEG. Cells were simultaneously stained with annexin V-FITC and propidium iodide (PI) using the FITC Annexin V Apoptosis Detection Kit (BD Pharmigen™). Dual fluorescence dot plots were determined with a FACSCalibur flow cytometer equipped with CellQuest software (BD Biosciences).

### Microarray experiments

SiHa_*parental*_ and SiHa_*CDV*_ cells were allowed to grow for 72 h in medium without CDV. Total RNA of 1 × 10^6^ cells was isolated with TRIzol reagent (Invitrogen) according to the manufacturer’s instructions. The RNA was further purified by RNeasy Mini Kit (Qiagen). RNA quality and quantity were assessed by using a Bioanalyzer system (Agilent).

Human Genome U133 Plus 2.0 arrays (Affymetrix) containing more than 54,000 probe sets and covering approximately 38,500 genes were used to analyze the expression profile of the two cell lines, and both conditions were tested in triplicate. Array hybridization, scanning and image analyzing were done following the manufacturer’s protocols (Affymetrix GeneChip Expression Assay) at the VIB Nucleomics Core Facility (http://www.nucleomics.be). Raw data were corrected for background signal using the RMA (Robust Multi-array analysis) algorithm (affy_1.22.0 package of BioConductor). The detection (Present/Absent) call generated by the Affymetrix microarray suite version 5 software (MAS 5.0) was used to remove probe sets that were not reliable detected in any of the microarrays before further analysis.

Differentially expressed (DE) probe sets between SiHa_*parental*_ and SiHa_*CDV*_ were determined using a moderated t-statistic test [LIMMA (linear models for microarray data), BioConductor]. The Benjamini-Hochberg correction for multiple testing [p < 0.05, false discovery rate (FDR) = 0.05] was performed. Probe sets were considered significantly DE if the absolute fold-change (FC) was > 2 and the P-value was < 0.05 (LIMMA) after applying the Benjamini-Hochberg correction.

The entire set of microarray data is deposited in the Gene Expression Omnibus (GEO, http://www.ncbi.nlm.nih.gov/projects/geo) according to MIAME standards under accession number GSE26748: http://www.ncbi.nlm.nih.gov/geo/query/acc.cgi?token=lpivfquymowyazo&acc=GSE26748.

Bioinformatics analysis of differentially expressed genes was carried out with Ingenuity Pathways Analysis (IPA, Ingenuity® Systems) version 9. Data sets with the corresponding FC and P-value were uploaded into the IPA (Ingenuity Pathway Analysis, Ingenuity® Systems) software. Stringent criteria, equivalent to those described for the selection of DE probes, were applied to identify DE genes. When genes were represented by 2 or more probe sets on the arrays, only the maximum FC was used. Uncharacterized probe sets were not included in the analysis.

The IPA application reveals relevant pathways and biological functions by comparing the number of genes that participate in a given function or pathway, relative to the total number of occurrences of those genes in all the pathways stored in the IPKB (Ingenuity Pathway Knowledge Base). Validation of the microarray data was performed with 4 genes (randomly selected) by quantitative reverse transcription-polymerase chain reaction (qPCR) as previously reported [28].

### Animal experiments

Female nu/nu NMRI mice (4–5 weeks old) were purchased from Janvier Breeding Center. All animal work was approved by the KU Leuven Ethics Committee for Animal Care and Use (Permission number: P160-2008).

Mice were inoculated sub-cutaneously on the back with 2 × 10^6^ cells in a volume of 200 μl, week 0 being considered the time point of cell inoculation. To estimate body weight gain, mice were sacrificed weekly and tumors were excised, weighed and subtracted from the total body weight. Gain in body weight was calculated as the percentage of body weight gained compared to the mice weight at week 0.

Spleens from 3 mice per group were isolated at different weeks to determine the percentage of immune cell populations. Spleens were processed and splenocytes were stained with specific antibodies and analyzed by flow cytometry as described previously [24]. One mouse per group was euthanized weekly to collect various tissues for histopathological examination.

Total blood from 5 mice per group was collected in EDTA tubes at week 5 to perform hematology and blood chemistry testing at the University Hospitals Leuven, Department of Laboratory Medicine, Leuven, Belgium.

At various time points, tumor- and host-derived cytokines were quantified in the sera of mice (3 animals per group) with a Bio-plex 200 system (Bio-Rad Laboratories) according to the manufacturer’s protocols.

### Statistical analysis

Statistical significance was assessed based on unpaired two-tailed Student’s *t*-test with GraphPad Prism 5 software (GraphPad Software Inc., La Jolla, CA, USA). Significance was indicated as: ns, not significant (p > 0.05); *, significant (p < values 0.05); **, very significant (p < 0.01); and ***, extremely significant (p < 0.001).

## Competing interests

The authors declare they have no competing interests.

## Authors’ contributions

TDS, GA, DT, PM, and RS conceived and designed the experiments. TDS, SD, and TM performed the experiments. TDS, GA, DT, JvdO, PM, and RS analyzed the data. TDS, GA, DT, and RS were responsible for drafting the article. All authors critically revised the article and finally approved the manuscript prior to publication.

## Supplementary Material

Additional file 1 **Validation of gene expression between microarray and qPCR.** The gene expression levels of microarray are presented by log_2_ fold changes, whereas those of qPCR are indicated by ∆∆Ct that are comparable to the log_2_ fold change values in microarray. DHRS2 (Dehydrogenase/reductase SDR family member 2), HIST1H2AC (Histone H2A type 1-C), GALC (Galactocerebrosidase), KRT14 (Keratin 14). Click here for file

Additional file 2 **Functional annotations associated with inflammatory response found to be distinct between SiHa**_***parental ***_**and SiHa**_***CDV***_**.** The criteria for selection of functional annotations were based on z-score and statistical significance (*P*-value < 0.05). The regulation z-score predicts whether an identified biological function is activated or inhibited. Positive z-scores indicate activation of a biological function, while negative z-scores suggest an inhibition. Absolute z-score values above 1 were considered significant. Click here for file

Additional file 3 **Canonical pathways related to immune response found to be distinct between SiHa**_***parental ***_**and SiHa**_***CDV***_**.** The significance of the associations between the genes from the two data sets and the canonical pathways were determined based on two parameters: (a) the *P*-value, calculated by the Fischer’s exact test, that determines the probability that there is an association between the genes in the data set and the canonical pathway that cannot be explained by chance alone and (b) the ratio of the number of genes from the data set in a given pathway divided by the total number of molecules in the given canonical pathway. *P*-values < 0.05 were considered statistically significant. Click here for file

Additional file 4 **Gene expression changes related to the ‘inflammatory response’ function in SiHa**_***CDV ***_**compared to SiHa**_***parental***_**.** Genes were considered significantly differentially expressed if the absolute fold-change (FC) was > 2 and the P-value was < 0.05 (LIMMA) after applying the Benjamini-Hochberg correction. Upregulated and downregulated genes are indicated by respectively positive and negative log_2_ fold changes. Click here for file

Additional file 5 **Effects of intratumoral CDV treatment on tumor growth in xenograft model.** (A) Kinetics of tumor growth were determined in mice bearing a xenograft that received either no treatment or intratumoral CDV treatment (25 μl of a 10 mg/ml CDV solution) once a day, five times per week, for a period of four weeks. Data are presented as the average tumor volume [mm^3^] of five mice (±SEM). Tumors were measured by means of a digital caliper in two directions (perpendicular diameters) and the formula V = (4πab^2^)/3, with ‘a’ and ‘b’ being the largest and smallest radius of the tumor, respectively, was applied to calculate the tumor volume. (B) Efficacy of treatment following four weeks of intratumoral CDV treatment. Efficacy was evaluated by means of the inhibitory rate (IR) on tumor growth and was calculated as IR = (C-T)/C x 100% (C and T being the respective tumor volumes of the untreated controls and the treated tumors). Treatment of SiHa_*parental*_ xenografts resulted in an IR of 95% (p-value < 0.01), while treatment of SiHa_*CDV*_ xenograft showed an IR of 51% (p-value > 0.05). Click here for file

Additional file 6 **Interleukin 6 (IL-6) levels in SiHa**_***parental ***_**and SiHa**_***CDV ***_**cell culture supernatants.** IL-6 levels were measured using the enzyme-linked immunosorbent assay kit (Invitrogen™) using cell culture supernatants. Cells were seeded at a density of 4 x 10^4^ cells per well in 3 ml culture medium in 6-well plates. After 24 h the medium was changed, and supernatant was subsequently collected after 3, 5, and 7 days. Human IL-6 levels in the supernatant (diluted 1:25 in PBS) of cultured cells were determined by using the IL-6 human ELISA kit following manufacturer’s instructions. Samples were measured in triplicate. Click here for file

Additional file 7 **STR profile of SiHa**_***parental ***_**and SiHa**_***CDV ***_**cells.** The different alleles for the STR loci that were identified in both cells lines are shown in the table. Determination of the STR profile of both cell lines illustrated a drift of a few markers (i.e. D1S1656, D21S11 and D1S1677) following long-term culturing of the cells. Overall these data demonstrated the relationship between the two cell lines and confirmed SiHa_*CDV*_ being a derivative of the SiHa_*parental*_ cells. Click here for file
