# Supplementary figures and images for: Reduced tumorigenicity and pathogenicity of cervical carcinoma SiHa cells selected for resistance to cidofovir
Source: Mol Cancer. 2013 Dec 10;12:158. doi: 10.1186/1476-4598-12-158 (PMC4029382; doi:10.1186/1476-4598-12-158)

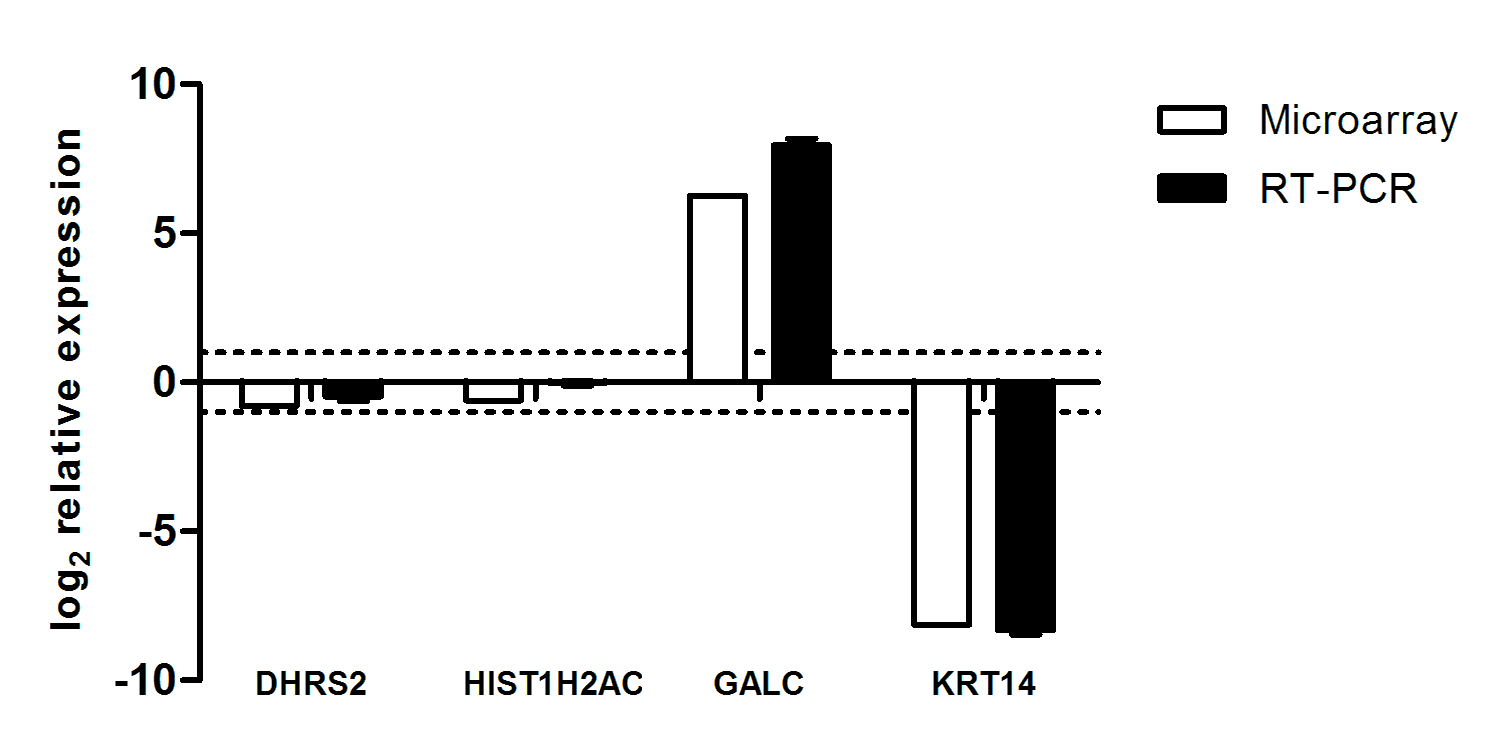

Supplement: Additional file 1 — Validation of gene expression between microarray and qPCR. The gene expression levels of microarray are presented by log2 fold changes, whereas those of qPCR are indicated by ∆∆Ct that are comparable to the log2 fold change values in microarray. DHRS2 (Dehydrogenase/reductase SDR family member 2), HIST1H2AC (Histone H2A type 1-C), GALC (Galactocerebrosidase), KRT14 (Keratin 14). [file 1476-4598-12-158-S1.tiff]

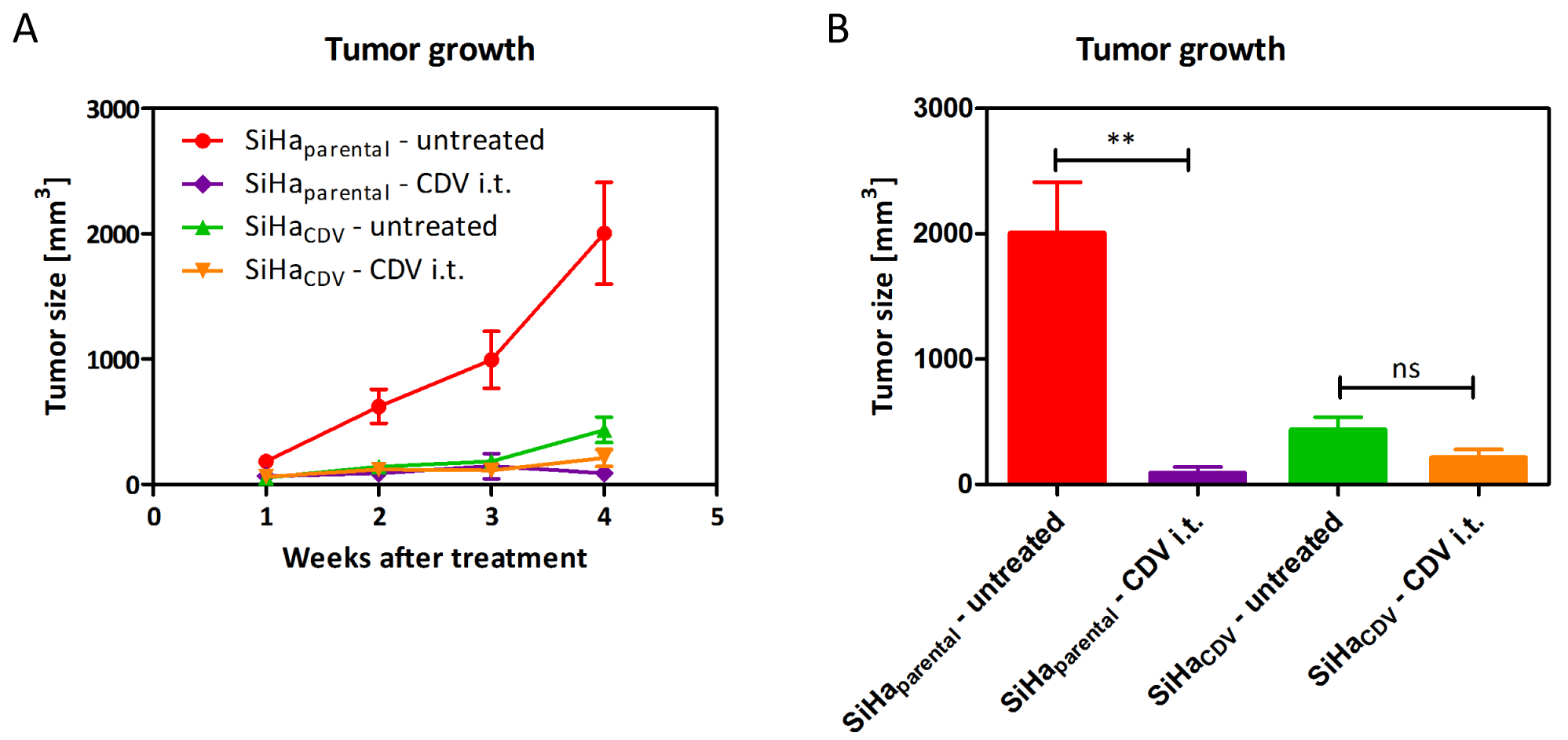

Supplement: Additional file 5 — Effects of intratumoral CDV treatment on tumor growth in xenograft model. (A) Kinetics of tumor growth were determined in mice bearing a xenograft that received either no treatment or intratumoral CDV treatment (25 μl of a 10 mg/ml CDV solution) once a day, five times per week, for a period of four weeks. Data are presented as the average tumor volume [mm3] of five mice (±SEM). Tumors were measured by means of a digital caliper in two directions (perpendicular diameters) and the formula V = (4πab2)/3, with ‘a’ and ‘b’ being the largest and smallest radius of the tumor, respectively, was applied to calculate the tumor volume. (B) Efficacy of treatment following four weeks of intratumoral CDV treatment. Efficacy was evaluated by means of the inhibitory rate (IR) on tumor growth and was calculated as IR = (C-T)/C x 100% (C and T being the respective tumor volumes of the untreated controls and the treated tumors). Treatment of SiHaparental xenografts resulted in an IR of 95% (p-value < 0.01), while treatment of SiHaCDV xenograft showed an IR of 51% (p-value > 0.05). [file 1476-4598-12-158-S5.tiff]

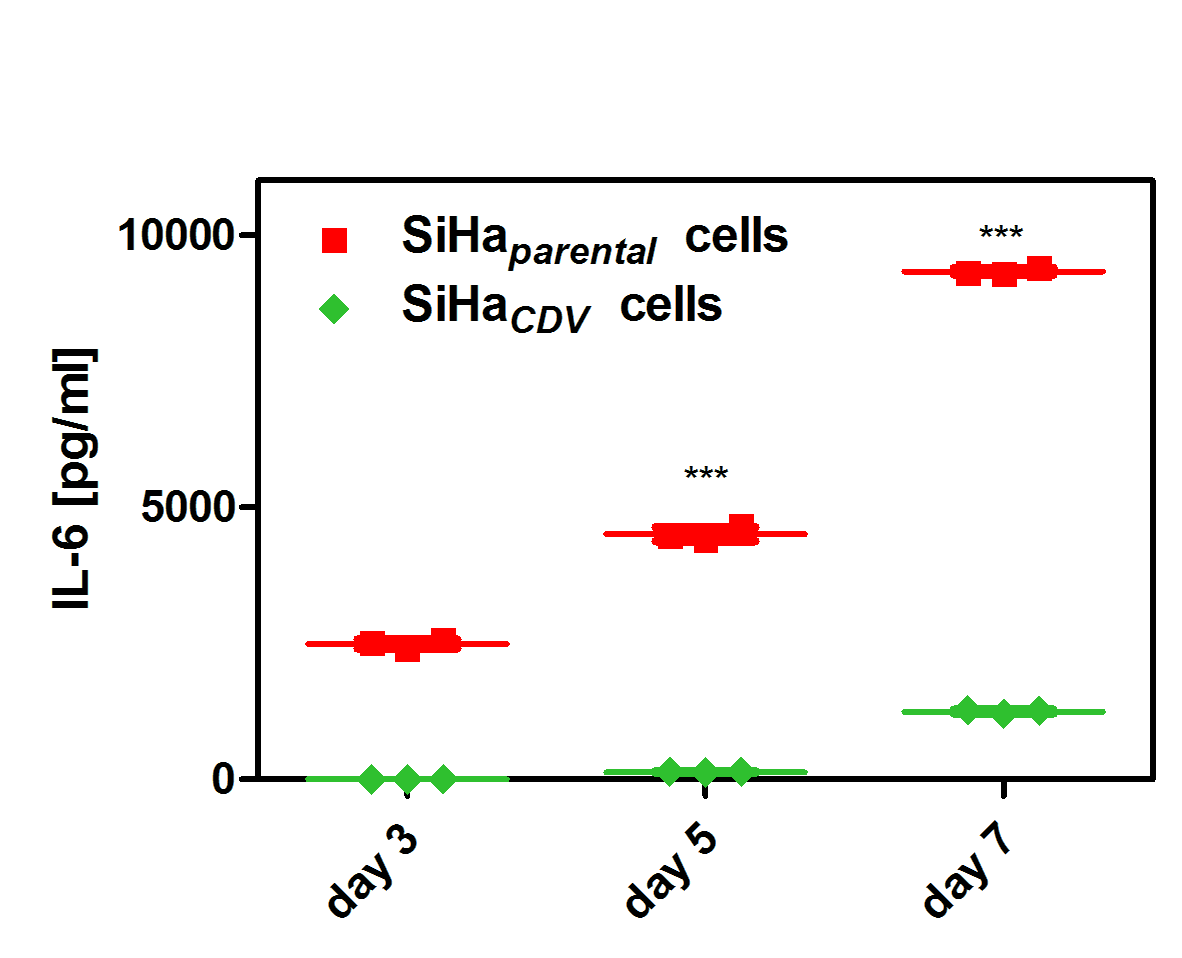

Supplement: Additional file 6 — Interleukin 6 (IL-6) levels in SiHaparental and SiHaCDV cell culture supernatants. IL-6 levels were measured using the enzyme-linked immunosorbent assay kit (Invitrogen™) using cell culture supernatants. Cells were seeded at a density of 4 x 10^4^ cells per well in 3 ml culture medium in 6-well plates. After 24 h the medium was changed, and supernatant was subsequently collected after 3, 5, and 7 days. Human IL-6 levels in the supernatant (diluted 1:25 in PBS) of cultured cells were determined by using the IL-6 human ELISA kit following manufacturer’s instructions. Samples were measured in triplicate. [file 1476-4598-12-158-S6.tiff]
