# Supplementary material for: Reduced tumorigenicity and pathogenicity of cervical carcinoma SiHa cells selected for resistance to cidofovir
Source: Mol Cancer. 2013 Dec 10;12:158. doi: 10.1186/1476-4598-12-158 (PMC4029382; doi:10.1186/1476-4598-12-158)
Supplement: Additional file 2 — Functional annotations associated with inflammatory response found to be distinct between SiHaparental and SiHaCDV. The criteria for selection of functional annotations were based on z-score and statistical significance (P-value < 0.05). The regulation z-score predicts whether an identified biological function is activated or inhibited. Positive z-scores indicate activation of a biological function, while negative z-scores suggest an inhibition. Absolute z-score values above 1 were considered significant. [file 1476-4598-12-158-S2.docx]

**Additional file 2.** **Functional annotations associated with inflammatory response found to be distinct between SiHa*_parental_* and SiHa*_CDV_* cells**.

| Functions Annotation | *P*-Value | Predicted Activation State | Regulation z-score | # Genes |
| --- | --- | --- | --- | --- |
| inflammatory response | 1,94E-08 | Decreased | -2,627 | 80 |
| activation of granulocytes | 2,62E-04 | Decreased | -2,844 | 17 |
| inflammation of organ | 4,54E-04 | Decreased | -2,307 | 32 |
| activation of neutrophils | 5,62E-04 | Decreased | -2,973 | 14 |

The criteria for selection of functional annotations were based on z-score and statistical significance (*P*-value < 0.05). The regulation z-score predicts whether an identified biological function is activated or inhibited. Positive z-scores indicate activation of a biological function, while negative z-scores suggest an inhibition. Absolute z-score values above 1 are considered significant.
