# Supplementary material for: Reduced tumorigenicity and pathogenicity of cervical carcinoma SiHa cells selected for resistance to cidofovir
Source: Mol Cancer. 2013 Dec 10;12:158. doi: 10.1186/1476-4598-12-158 (PMC4029382; doi:10.1186/1476-4598-12-158)
Supplement: Additional file 3 — Canonical pathways related to immune response found to be distinct between SiHaparental and SiHaCDV. The significance of the associations between the genes from the two data sets and the canonical pathways were determined based on two parameters: (a) the P-value, calculated by the Fischer’s exact test, that determines the probability that there is an association between the genes in the data set and the canonical pathway that cannot be explained by chance alone and (b) the ratio of the number of genes from the data set in a given pathway divided by the total number of molecules in the given canonical pathway. P-values < 0.05 were considered statistically significant. [file 1476-4598-12-158-S3.docx]

**Additional file 3.** **Canonical pathways related to immune response** **found to be distinct between SiHa*_parental_* and SiHa*_CDV_* cells.**

| Ingenuity Canonical Pathways | -log(*P*-value)^a^ | Ratio^b^ |
| --- | --- | --- |
| PI3K Signaling in B Lymphocytes | 4.52E+00 | 0.15 (22/147) |
| Acute Phase Response Signaling | 4.43E+00 | 0.15 (26/178) |
| Role of Pattern Recognition Receptors in Recognition of Bacteria and Viruses | 3.56E+00 | 0.16 (14/87) |
| IL-9 Signaling | 2.85E+00 | 0.20 (8/40) |
| NF-κB Signaling | 2.74E+00 | 0.12 (21/176) |
| HMGB1 Signaling | 2.49E+00 | 0.14 (14/100) |
| Role of PKR in Interferon Induction and Antiviral Response | 2.37E+00 | 0.17 (8/46) |
| Complement System | 2.36E+00 | 0.20 (7/35) |
| Toll-like Receptor Signaling | 2.35E+00 | 0.16 (9/55) |
| CD27 Signaling in Lymphocytes | 2.23E+00 | 0.16 (9/57) |
| Production of Nitric Oxide and Reactive Oxygen Species in Macrophages | 2.13E+00 | 0.10 (21/210) |
| IL-6 Signaling | 2.04E+00 | 0.13 (13/100) |
| Regulation of IL-2 Expression in Activated and Anergic T Lymphocytes | 1.99E+00 | 0.12 (11/89) |
| IL-10 Signaling | 1.86E+00 | 0.13 (10/78) |
| Interferon Signaling | 1.76E+00 | 0.17 (6/36) |
| CTLA4 Signaling in Cytotoxic T Lymphocytes | 1.64E+00 | 0.11 (11/98) |
| MSP-RON Signaling Pathway | 1.61E+00 | 0.14 (7/51) |
| B Cell Receptor Signaling | 1.60E+00 | 0.10 (16/156) |
| Clathrin-mediated Endocytosis Signaling | 1.51E+00 | 0.10 (19/195) |
| IL-8 Signaling | 1.45E+00 | 0.09 (18/193) |
| IL-1 Signaling | 1.42E+00 | 0.10 (11/107) |

The significance of the associations between the genes from the two data sets and the canonical pathways were determined based on two parameters: (a) the *P*-value, calculated by the Fischer’s exact test, that determines the probability that there is an association between the genes in the data set and the canonical pathway that cannot be explained by chance alone and (b) the ratio of the number of genes from the data set in a given pathway divided by the total number of molecules in the given canonical pathway. *P*-values < 0.05 were considered statistically significant.
