# Supplementary material for: Reduced tumorigenicity and pathogenicity of cervical carcinoma SiHa cells selected for resistance to cidofovir
Source: Mol Cancer. 2013 Dec 10;12:158. doi: 10.1186/1476-4598-12-158 (PMC4029382; doi:10.1186/1476-4598-12-158)
Supplement: Additional file 4 — Gene expression changes related to the ‘inflammatory response’ function in SiHaCDV compared to SiHaparental. Genes were considered significantly differentially expressed if the absolute fold-change (FC) was > 2 and the P-value was < 0.05 (LIMMA) after applying the Benjamini-Hochberg correction. Upregulated and downregulated genes are indicated by respectively positive and negative log2 fold changes. [file 1476-4598-12-158-S4.docx]

**Additional file 4. Gene expression changes related to the ‘inflammatory response’ function in SiHa*_CDV_* compared to SiHa*_parental_* cells.**

Genes were considered significantly differentially expressed if the absolute fold-change (FC) was > 2 and the P-value was < 0.05 (LIMMA) after applying the Benjamini-Hochberg correction. Upregulated and downregulated genes are indicated by respectively positive and negative log_2_ fold changes.

| **Symbol** | **Entrez Gene Name** | **Log_2_ fold** |
| --- | --- | --- |
| ADAM12 | ADAM metallopeptidase domain 12 | **1,85** |
| AKT3 | v-akt murine thymoma viral oncogene homolog 3 (protein kinase B, gamma) | **-2,22** |
| AOX1 | aldehyde oxidase 1 | **-3,52** |
| APRT | adenine phosphoribosyltransferase | **-1,24** |
| ARAP3 | ArfGAP with RhoGAP domain, ankyrin repeat and PH domain 3 | **-1,29** |
| ARHGDIB | Rho GDP dissociation inhibitor (GDI) beta | **-2,67** |
| ARRB1 | arrestin, beta 1 | **-1,62** |
| ATG5 | autophagy related 5 | **1,15** |
| AXL | AXL receptor tyrosine kinase | **1,12** |
| BID | BH3 interacting domain death agonist | **-1,10** |
| C1R | complement component 1, r subcomponent | **2,26** |
| C3 | complement component 3 | **-3,90** |
| C5 | complement component 5 | **-2,06** |
| C5AR1 | complement component 5a receptor 1 | **-3,57** |
| CA13 | carbonic anhydrase XIII | **-1,93** |
| CALB1 | calbindin 1, 28kDa | **-4,21** |
| CAMK1D | calcium/calmodulin-dependent protein kinase ID | **-1,21** |
| CAV1 | caveolin 1, caveolae protein, 22kDa | **-1,04** |
| CBLB | Cbl proto-oncogene, E3 ubiquitin protein ligase B | **1,46** |
| CCL2 | chemokine (C-C motif) ligand 2 | **-2,20** |
| CCL26 | chemokine (C-C motif) ligand 26 | **-1,14** |
| CCL5 | chemokine (C-C motif) ligand 5 | **1,83** |
| CCND1 | cyclin D1 | **2,18** |
| CCR3 | chemokine (C-C motif) receptor 3 | **1,45** |
| CD14 | CD14 molecule | **-2,75** |
| CD24 | CD24 molecule | **-2,67** |
| CD44 | CD44 molecule (Indian blood group) | **1,27** |
| CD47 | CD47 molecule | **1,16** |
| CD55 | CD55 molecule, decay accelerating factor for complement (Cromer blood group) | **1,18** |
| CD9 | CD9 molecule | **-1,04** |
| CEBPB | CCAAT/enhancer binding protein (C/EBP), beta | **1,81** |
| CEBPE | CCAAT/enhancer binding protein (C/EBP), epsilon | **-1,77** |
| CFH | complement factor H | **-2,55** |
| CLU | clusterin | **1,77** |
| CTGF | connective tissue growth factor | **2,41** |
| CTSL1 | cathepsin L1 | **1,50** |
| CXADR | coxsackie virus and adenovirus receptor | **-1,01** |
| CXCL1 | chemokine (C-X-C motif) ligand 1 (melanoma growth stimulating activity, alpha) | **1,51** |
| CXCL17 | chemokine (C-X-C motif) ligand 17 | **-3,50** |
| CXCL2 | chemokine (C-X-C motif) ligand 2 | **2,53** |
| CXCR7 | chemokine (C-X-C motif) receptor 7 | **1,12** |
| DAB2 | disabled homolog 2, mitogen-responsive phosphoprotein (Drosophila) | **1,79** |
| DEFB1 | defensin, beta 1 | **-1,35** |
| EDN1 | endothelin 1 | **-1,37** |
| EDNRB | endothelin receptor type B | **-5,29** |
| EGLN3 | egl nine homolog 3 (C. elegans) | **-1,29** |
| EIF2AK2 | eukaryotic translation initiation factor 2-alpha kinase 2 | **1,39** |
| EPHA4 | EPH receptor A4 | **1,28** |
| ETS1 | v-ets erythroblastosis virus E26 oncogene homolog 1 (avian) | **-1,56** |
| F11R | F11 receptor | **-4,44** |
| F12 | coagulation factor XII (Hageman factor) | **-1,28** |
| F3 | coagulation factor III (thromboplastin, tissue factor) | **1,11** |
| FKBP1A | FK506 binding protein 1A, 12kDa | **-1,53** |
| FOS | FBJ murine osteosarcoma viral oncogene homolog | **-1,57** |
| FOXF1 | forkhead box F1 | **1,24** |
| FUT4 | fucosyltransferase 4 (alpha (1,3) fucosyltransferase, myeloid-specific) | **1,10** |
| FYN | FYN oncogene related to SRC, FGR, YES | **2,83** |
| GAL | galanin prepropeptide | **4,20** |
| GCNT1 | glucosaminyl (N-acetyl) transferase 1, core 2 | **-1,79** |
| GNAQ | guanine nucleotide binding protein (G protein), q polypeptide | **1,35** |
| GNAS | GNAS complex locus | **-1,12** |
| GSN | gelsolin | **1,98** |
| HDAC4 | histone deacetylase 4 | **1,00** |
| HLA-DMA | major histocompatibility complex, class II, DM alpha | **-1,27** |
| HMOX1 | heme oxygenase (decycling) 1 | **1,50** |
| HSPD1 | heat shock 60kDa protein 1 (chaperonin) | **-1,36** |
| IFI44 | interferon-induced protein 44 | **2,63** |
| IFI44L | interferon-induced protein 44-like | **1,92** |
| IFI6 | interferon, alpha-inducible protein 6 | **2,12** |
| IFIT1 | interferon-induced protein with tetratricopeptide repeats 1 | **1,20** |
| IGF1R | insulin-like growth factor 1 receptor | **1,39** |
| IK | IK cytokine, down-regulator of HLA II | **-1,32** |
| IKBKG | inhibitor of kappa light polypeptide gene enhancer in B-cells, kinase gamma | **1,53** |
| IL11 | interleukin 11 | **1,18** |
| IL15 | interleukin 15 | **-1,05** |
| IL18R1 | interleukin 18 receptor 1 | **1,85** |
| IL1R1 | interleukin 1 receptor, type I | **1,50** |
| IL1RL1 | interleukin 1 receptor-like 1 | **1,36** |
| IL20 | interleukin 20 | **1,29** |
| IL27RA | interleukin 27 receptor, alpha | **-1,51** |
| IL7R | interleukin 7 receptor | **-1,93** |
| IRAK3 | interleukin-1 receptor-associated kinase 3 | **1,28** |
| IRF7 | interferon regulatory factor 7 | **1,39** |
| IRF9 | interferon regulatory factor 9 | **2,77** |
| ITCH | itchy E3 ubiquitin protein ligase | **1,05** |
| ITGA6 | integrin, alpha 6 | **-1,17** |
| ITGB2 | integrin, beta 2 (complement component 3 receptor 3 and 4 subunit) | **-1,11** |
| KCNN4 | potassium intermediate/small conductance calcium-activated channel, subfamily N, member 4 | **-1,62** |
| KLF4 | Kruppel-like factor 4 (gut) | **1,41** |
| KRT8 | keratin 8 | **-5,02** |
| LAMP2 | lysosomal-associated membrane protein 2 | **1,64** |
| LCAT | lecithin-cholesterol acyltransferase | **1,35** |
| LEPR | leptin receptor | **-2,81** |
| LGALS1 | lectin, galactoside-binding, soluble, 1 | **1,29** |
| LIAS | lipoic acid synthetase | **-1,39** |
| MAP3K5 | mitogen-activated protein kinase kinase kinase 5 | **1,21** |
| MAVS | mitochondrial antiviral signaling protein | **1,09** |
| MBP | myelin basic protein | **-1,43** |
| MCAM | melanoma cell adhesion molecule | **2,72** |
| MDK | midkine (neurite growth-promoting factor 2) | **2,00** |
| MIB1 | mindbomb E3 ubiquitin protein ligase 1 | **1,19** |
| MREG | melanoregulin | **-1,52** |
| MUC1 | mucin 1, cell surface associated | **-1,59** |
| MX1 | myxovirus (influenza virus) resistance 1, interferon-inducible protein p78 (mouse) | **2,27** |
| MYLK | myosin light chain kinase | **-1,10** |
| NCF2 | neutrophil cytosolic factor 2 | **-1,21** |
| NFIL3 | nuclear factor, interleukin 3 regulated | **1,45** |
| NMU | neuromedin U | **2,59** |
| NOG | noggin | **-4,36** |
| NR4A2 | nuclear receptor subfamily 4, group A, member 2 | **1,94** |
| NR4A3 | nuclear receptor subfamily 4, group A, member 3 | **2,28** |
| NT5E | 5'-nucleotidase, ecto (CD73) | **2,36** |
| NUPR1 | nuclear protein, transcriptional regulator, 1 | **1,25** |
| OAS1 | 2'-5'-oligoadenylate synthetase 1, 40/46kDa | **1,47** |
| PAFAH1B1 | platelet-activating factor acetylhydrolase 1b, regulatory subunit 1 (45kDa) | **1,05** |
| PAG1 | phosphoprotein associated with glycosphingolipid microdomains 1 | **-1,05** |
| PECAM1 | platelet/endothelial cell adhesion molecule 1 | **-1,29** |
| PIK3R1 | phosphoinositide-3-kinase, regulatory subunit 1 (alpha) | **-1,01** |
| PLA2G4A | phospholipase A2, group IVA (cytosolic, calcium-dependent) | **-2,32** |
| PLAA | phospholipase A2-activating protein | **1,18** |
| PLD1 | phospholipase D1, phosphatidylcholine-specific | **-1,53** |
| PLSCR1 | phospholipid scramblase 1 | **1,67** |
| POLR3G | polymerase (RNA) III (DNA directed) polypeptide G (32kD) | **-1,52** |
| PRDM1 | PR domain containing 1, with ZNF domain | **-3,10** |
| PREX1 | phosphatidylinositol-3,4,5-trisphosphate-dependent Rac exchange factor 1 | **1,68** |
| PROCR | protein C receptor, endothelial | **-1,81** |
| PROK2 | prokineticin 2 | **1,71** |
| PROS1 | protein S (alpha) | **-1,37** |
| PSCA | prostate stem cell antigen | **-2,23** |
| PTGS1 | prostaglandin-endoperoxide synthase 1 (prostaglandin G/H synthase and cyclooxygenase) | **-1,75** |
| PTK2 | PTK2 protein tyrosine kinase 2 | **1,46** |
| PTX3 | pentraxin 3, long | **1,28** |
| RAB27A | RAB27A, member RAS oncogene family | **-1,18** |
| RAB27B | RAB27B, member RAS oncogene family | **1,19** |
| RHOB | ras homolog family member B | **1,55** |
| S100A14 | S100 calcium binding protein A14 | **-1,75** |
| SECTM1 | secreted and transmembrane 1 | **1,88** |
| SERPINA1 | serpin peptidase inhibitor, clade A (alpha-1 antiproteinase, antitrypsin), member 1 | **-1,91** |
| SERPINB9 | serpin peptidase inhibitor, clade B (ovalbumin), member 9 | **4,12** |
| SERPINE1 | serpin peptidase inhibitor, clade E (nexin, plasminogen activator inhibitor type 1), member 1 | **-1,36** |
| SERPINE2 | serpin peptidase inhibitor, clade E (nexin, plasminogen activator inhibitor type 1), member 2 | **1,46** |
| SH2D2A | SH2 domain containing 2A | **-1,12** |
| SOCS2 | suppressor of cytokine signaling 2 | **-1,19** |
| SOCS3 | suppressor of cytokine signaling 3 | **-1,49** |
| SPHK1 | sphingosine kinase 1 | **1,70** |
| SRGN | serglycin | **-1,75** |
| STAT1 | signal transducer and activator of transcription 1, 91kDa | **1,09** |
| STAT3 | signal transducer and activator of transcription 3 (acute-phase response factor) | **-1,26** |
| STEAP2 | STEAP family member 2, metalloreductase | **-1,77** |
| STXBP1 | syntaxin binding protein 1 | **1,83** |
| TFF1 | trefoil factor 1 | **-1,05** |
| TGFB1 | transforming growth factor, beta 1 | **-1,93** |
| TGM2 | transglutaminase 2 (C polypeptide, protein-glutamine-gamma-glutamyltransferase) | **-2,02** |
| TIAM1 | T-cell lymphoma invasion and metastasis 1 | **-2,04** |
| TIMP2 | TIMP metallopeptidase inhibitor 2 | **1,15** |
| TIMP3 | TIMP metallopeptidase inhibitor 3 | **-1,72** |
| TLR3 | toll-like receptor 3 | **-1,65** |
| TLR4 | toll-like receptor 4 | **-2,01** |
| TNFAIP6 | tumor necrosis factor, alpha-induced protein 6 | **1,07** |
| TNFRSF11B | tumor necrosis factor receptor superfamily, member 11b | **1,42** |
| TNFRSF21 | tumor necrosis factor receptor superfamily, member 21 | **1,14** |
| TNFSF15 | tumor necrosis factor (ligand) superfamily, member 15 | **-2,06** |
| TNFSF9 | tumor necrosis factor (ligand) superfamily, member 9 | **-1,68** |
| TOP2A | topoisomerase (DNA) II alpha 170kDa | **1,02** |
| TRAF3 | TNF receptor-associated factor 3 | **1,26** |
| TRIM22 | tripartite motif containing 22 | **-1,19** |
| TUB | tubby homolog (mouse) | **1,41** |
| UACA | uveal autoantigen with coiled-coil domains and ankyrin repeats | **-1,07** |
| VAV3 | vav 3 guanine nucleotide exchange factor | **-2,82** |
| VDR | vitamin D (1,25- dihydroxyvitamin D3) receptor | **-2,08** |
| VTN | vitronectin | **1,56** |
| XPR1 | xenotropic and polytropic retrovirus receptor 1 | **1,11** |
| ZEB1 | zinc finger E-box binding homeobox 1 | **2,15** |
