# Supplementary material for: Reduced tumorigenicity and pathogenicity of cervical carcinoma SiHa cells selected for resistance to cidofovir
Source: Mol Cancer. 2013 Dec 10;12:158. doi: 10.1186/1476-4598-12-158 (PMC4029382; doi:10.1186/1476-4598-12-158)
Supplement: Additional file 7 — STR profile of SiHaparental and SiHaCDV cells. The different alleles for the STR loci that were identified in both cells lines are shown in the table. Determination of the STR profile of both cell lines illustrated a drift of a few markers (i.e. D1S1656, D21S11 and D1S1677) following long-term culturing of the cells. Overall these data demonstrated the relationship between the two cell lines and confirmed SiHaCDV being a derivative of the SiHaparental cells. [file 1476-4598-12-158-S7.docx]

**Additional file 7. STR profile of SiHa*_parental_* and SiHa*_CDV_* cells.**

| STR Locus | SiHa*_parental_* | SiHa*_CDV_* |
| --- | --- | --- |
| D2S441 | 10 | 10 |
| D1S1656 | 14-15-16 | 14-15 |
| D12S391 | 19-22 | 19-22 |
| D10S1248 | 13-17 | 13-17 |
| D21S11 | 29-31-32 | 29-31 |
| D22S1045 | 12 | 12 |
| AMEL | X | X |
| D18S51 | 15 | 15 |
| D1S1677 | 14 | 12-14 |
| FGA | 21 | 21 |

The different alleles for the STR loci that were identified in both cells lines are shown in the table. Determination of the STR profile of both cell lines illustrated a drift of a few markers (i.e. D1S1656, D21S11 and D1S1677) following long-term culturing of the cells. Overall these data demonstrated the relationship between the two cell lines and confirmed SiHa*_CDV_* being a derivative of the SiHa*_parental_* cells.
